# The Rewards of French Army Surgeons

**Published:** 1859-01

**Authors:** 


*The Rewards of French Army Surgeons.*—We find the following in the *Moniteur*:—The *Presse*, in its number of the 23d ult., published, in the name of several army surgeons who had served, during the late war in the East, in the hospitals and ambulances of Constantinople, Varna, and Dobrudscha, a claim, that the medal commemorative of the campaign in the Crimea, should be awarded them. The Minister-of-War has never underrated the services rendered by that portion of the Medical attendants in the army, and, on all occasions, he has taken a pleasure in rendering it the most striking justice. But, according to the intentions forcibly expressed by the British Government, the English medal was to be *exclusively* given, both in the English and in the French army, to the soldiers having served in the Crimea betwen the 14th of September, 1854, the day of the landing at Old Fort, and the taking of Sebastopol, on the 8th of September, 1855. All the members of the medical corps who fulfilled this condition, received, like all the other corps of the army, the Crimean medal as well as the clasps indicating the engagements at which they were present. It was not possible to include the medical officers who had not served in the Crimea in the distribution, which was regulated by an international convention, and effected on an invariable rule, which was not once departed from, even in favor of the troops landed in the Crimea, a very short time after the taking of Sebastopol.

